# Protocol and statistical analysis plan for a randomized controlled trial of the effect of intravenous iron on anemia in Malawian pregnant women in their third trimester (REVAMP – TT)

**DOI:** 10.12688/gatesopenres.14710.2

**Published:** 2023-12-18

**Authors:** Rebecca Harding, Ernest Moya, Ricardo Ataíde, Zinenani Truwah, Glory Mzembe, Gomezgani Mhango, Ayşe V. Demir, William Stones, Louise Randall, Marc Seal, Katherine Johnson, Stefan Bode, Martin N. Mwangi, Sant-Rayn Pasricha, Sabine Braat, Kamija S. Phiri

**Affiliations:** 1 Population Health and Immunity, Walter and Eliza Hall Institute of Medical Research, Parkville, Vic, 3052, Australia; 2 Training and Research Unit of Excellence (TRUE), 1 Kufa Road, P.O. Box 30538, Chichiri, Blantyre, BT3, Malawi; 3 Department of Public Health, Kamuzu University of Health Sciences, School of Global and Public Health, Private Bag 360, Chichiri, Blantyre, BT3, Malawi; 4 Infectious Diseases, Peter Doherty Institute for Infection and Immunity, Melbourne, VIC, 3000, Australia; 5 Meander Medical Center, Laboratory for Clinical Chemistry, Maatweg 3, Amersfoort, 3813 TZ, The Netherlands; 6 Centre for Reproductive Health, Kamuzu University of Health Sciences, Private Bag 360, Chichiri, Blantyre, BT3, Malawi; 7 Developmental Imaging, Murdoch Children's Research Institute, Parkville, Victoria, 3052, Australia; 8 Melbourne School of Psychological Sciences, The University of Melbourne, Melbourne, Victoria, 3010, Australia; 9 The Micronutrient Forum, Washington DC, 20005-5905, USA; 10 Diagnostic Haematology and Clinical Haematology, The Peter MacCallum Cancer Centre, The Royal Melbourne Hospital, Parkville, Victoria, 3052, Australia; 11 Centre for Epidemiology and Biostatistics, School of Population and Global Health,, The University of Melbourne, Melbourne, Victoria, 3000, Australia

**Keywords:** Pregnancy, Anemia, Iron, Iron-deficiency, Birth weight, RCT

## Abstract

**Background:**

Anemia affects 40% of pregnant women globally, leading to maternal mortality, premature birth, low birth weight, and poor baby development. Iron deficiency causes over 40% of anemia cases in Africa. Oral iron supplementation is insufficient for Low-and-Middle-Income-Countries (LMICs) to meet current WHO targets. We hypothesized that a single intravenous dose of Ferric Carboxymaltose (FCM) may be more effective than oral iron treatment for anemia recovery, particularly in these settings where women present late for antenatal care.

**Methods:**

This is a two-arm parallel open-label individual-randomized controlled trial in third trimester, in malaria Rapid Diagnostic Test-negative pregnant women with moderate or severe anemia - capillary hemoglobin <10 g/dL – who are randomized to receive either parenteral iron – with FCM – or standard-of-care oral iron for the remainder of pregnancy. This is the sister trial to the second-trimester **REVAMP** trial, funded by the Bill and Melinda Gates Foundation (trial registration ACTRN12618001268235, Gates Grant number INV-010612). In REVAMP-TT, recruitment and treatment are performed within primary health centers. The trial will recruit 590 women across Zomba district, Malawi. The primary outcome is the proportion of anemic women - venous hemoglobin <11 g/dL - at 36 weeks’ gestation or delivery (whichever occurs first). Other pre-specified key secondary clinical and safety outcomes include maternal iron-status and hypophosphatemia, neonate birth weight, infant growth and infant iron and hematological parameters.

**Discussion:**

This study will determine whether FCM, delivered within primary health centers, is effective, safe and feasible for treating moderate to severe anemia in third-trimester pregnant Malawian women. This intervention could have long-term benefits for maternal and child health, resulting in improved survival and child development.

## Strengths and limitations of this study

- This trial is conducted in primary health care centers.
- Addresses a population that is at high-risk of poor maternal and neonatal outcomes.
- Provides iron at a pregnancy stage when iron transfer to the fetus is maximum.
- Use of fundal height limits the accuracy of gestational age assessment.

## Introduction / background

Anemia during pregnancy is a critical global health problem, affecting almost 40% of pregnant women worldwide, with the highest rates being found in Africa and Asia ^1^. It is associated with significant risks for both the mother and the child, including maternal mortality, prematurity, low birth weight, and impaired development ^2– 4^. Control of anemia in women is a key 2025 global nutrition target ^5^.

Oral iron supplementation during pregnancy has been shown to be beneficial and safe ^6^. A trial in 470 Kenyan pregnant women demonstrated the benefits of oral iron supplementation in reducing the risk of low birth weight, lengthening gestation duration, and reducing the risk of premature birth ^7^. Global recommendations for the management of anemia in pregnancy in Low- and Middle-Income Countries (LMICs) are that women be treated with high dose of daily oral iron supplementation for three months (120 mg of elemental iron) ^8, 9^. However, uptake and adherence to oral iron therapy during pregnancy are inadequate ^10^. Moreover, even when delivered, oral iron frequently fails to correct anemia in routine practice ^11^. These challenges need to be addressed to ensure adequate uptake and adherence to oral therapy during pregnancy if the 2025 global nutrition target of a 50% reduction of anemia in women of reproductive age is to be achieved.

Ferric carboxymaltose (FCM) is an intravenous iron therapy that has revolutionized the treatment of iron deficiency anemia ^12^. It can be administered over 15 minutes and at doses up to 1000 mg, allowing for a total dose of iron replacement to be achieved in a single visit ^12^. FCM has been available in Europe since its approval in 2007 and in the USA since 2009, and over 50 countries currently market it ^12^. FCM has become a widely used treatment for anemia in pregnancy in developed countries ^13– 15^. However, little data exists on the feasibility, safety profile and clinical efficacy of delivering FCM in LMICs, including its effects on maternal anemia, and the effects on postpartum health or long-term infant growth and development.

In our two-arm open-label individual-randomized controlled trial (REVAMP) of moderate to severe anemic women in the second trimester of pregnancy in the Blantyre and Zomba district, Malawi, we found that compared to standard-of-care oral iron, FCM could be safely delivered in 862 women with no serious infusion reactions and no increase in adverse events ^16^. Although FCM reduced iron deficiency and iron deficiency anemia across timepoints from four weeks post infusion, week 36 gestation, delivery, and four weeks postpartum, a statistically significant reduction in anemia was not observed except for four weeks post-infusion (77% FCM versus 84% standard-of-care). The feasibility of implementing such an intervention within a primary care health setting is being explored within an associated project to ensure target populations that may benefit most can receive it ^17^. Our team conducted the REVAMP trial ^18, 19^, funded by the Bill and Melinda Gates Foundation (trial registration ACTRN12618001268235, Gates Grant number INV-010612), which was published this year ^16^. This trial looked at the safety and efficacy of delivering Ferric Carboxymaltose to anemic women in their second trimester of pregnancy, with the intervention being delivered at the research sites in Zomba and Blantyre, southern Malawi. At four weeks post-intervention women in the IV-iron group were significantly less anemic than those in the oral iron arm, but this was not carried to the primary outcome at 36 weeks gestation or had any significant impact in key outcomes such as maternal anemia at delivery and baby birth weight ^16^. Given that the third trimester is when fetal iron requirement reaches its peak, REVAMP-TT has the potential to target the most important period for iron availability during pregnancy ^20^.

In REVAMP-TT, we will determine the effectiveness of intravenous iron – given as FCM – once during the third trimester (27–35 weeks’ gestation) on anemia recovery by 36 weeks’ gestation or at delivery, whichever comes first, compared to standard-of-care oral iron. We hypothesize that in Malawian pregnant women with anemia in the third trimester, compared to standard-of-care, treatment with a single dose of FCM will be superior in terms of a reduction in anemia prevalence prior to or at delivery. We also hypothesize that FCM will have positive impacts on other maternal and neonatal outcomes and will be safe.

## Methods

### Ethics and dissemination

National Health Sciences Research Committee of Malawi Approval – NHSRC REF. Number: 20/11/2622. Human Research Ethics committee at WEHI – HRE REF. Number: 20/25.

### Trial registration number

Australia and New Zealand Clinical Trial Registry – ANZCTR 12621001239853.

### Protocol

This manuscript summarizes the trial protocol and statistical analysis plan (SAP).

**Figure 1.**
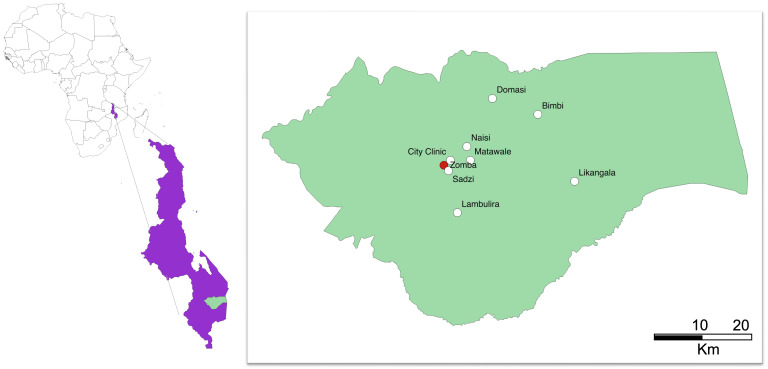
Map of Malawi with location of Zomba district, the coordinating trial site in Zomba (red) and the recruiting government primary health centers (white). Figure prepared in QGIS2.10.

**Figure 2.**
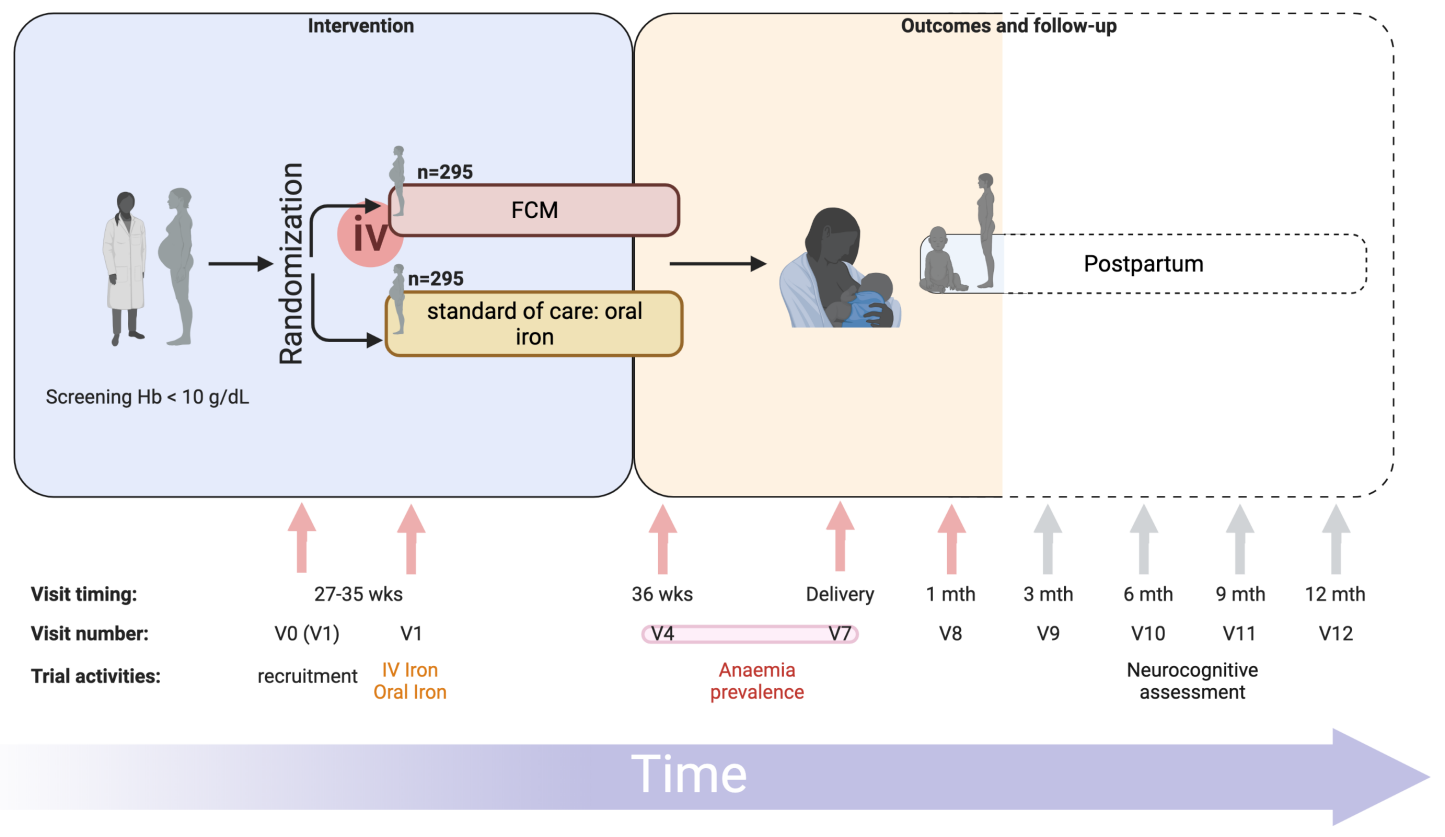
REVAMP-TT Trial schema. Trial design with visit timings, visit numbers (standardized to match those of REVAMP trial – P.02/18/2357) and main trial activities are represented. FCM, Ferric Carboxymaltose; iv, intravenous; mth, months; REVAMP-TT, Randomized controlled trial of the Effect of intraVenous iron on Anemia in Malawian Pregnant women - Third Trimester; V0, visit 0; V1, visit 1; V4, visit 4; V7, visit 7; V8, visit 8; V9, visit 9; V10, visit 10; V11, visit 11; V12, visit 12; wks, weeks. Primary outcome measured at V4 or V7, indicated by the red bar connecting both visits. Dotted lines represent follow-up visits not included in the main trial. Figure prepared using BioRender.

In short, pregnant women attending antenatal care at eight primary health centres in Zomba district, Malawi ( Figure 1), will be prescreened. Women in their third trimester, with negative malaria rapid diagnostic tests (RDT) and a capillary hemoglobin of 10 g/dL or less will be invited to be enrolled in the trial ( Figure 2). Women will be randomized to receive either intravenous FCM or standard-of-care oral iron. All trial procedures and follow-up times are detailed in Table 1. This protocol describes the main trial looking at pregnant women in their third trimester and following them until one-month postpartum, after which we will report on the primary and secondary objectives. Follow-up of mothers and infants from one-month postpartum to 12 months postpartum consists of a range of exploratory economic, biological, cognitive and clinical outcomes. These analyses are beyond the scope of this protocol, as are the exploratory outcomes collected up to 12-months postpartum and will be reported separately. The trial design is summarized in Figure 2.

**Table 1.** Trial visits and activities.

Protocol Activity	Prescreening	Enrolment Week 27–35	36 weeks ±2d (pre- delivery)	Delivery ±2d	28 days postpartum ± 2d	Unscheduled visits
Pre-screening form completion	X					
Screening	X					
Informed consent process		X				
Medical & obstetric history		X				
Household economic data form		X				
Household food insecurity			X			
Complete physical examination ^a^		X				
Limited physical examination ^b^			X	X	X	
Mother Infant Bonding Scale data forms completion						
Edinburgh Postpartum depression scale (EPDS)		X				
Participant arm allocation		X				
Administer treatment						
Intravenous iron		X				
Oral iron		X				
Laboratory procedures (Maternal)						
Full Blood Count (including Hb)		X	X	X	X	
Hemoglobin (capillary)	X					
Malaria RDT		X				X
Malaria microscopy		X	X	X	X	X
Malaria filter paper for PCR		X	X	X	X	X
Serum for iron markers tests ^c^		X	X	X	X	
Serum for inflammatory markers tests ^d^		X	X	X	X	
Phosphate		X	X	X	X	
Placenta histology				X		
Breast milk sample ^f^					X	
Laboratory procedures (Infant)						
Full Blood Count				X (cord)	X	
Malaria microscopy				X (cord)	X	
Serum for iron markers tests ^c^				X (cord)	X	
Malaria filter paper for PCR				X (cord)	X	
Stool sample ^e^					X	
Pregnancy outcome				X	X	
Birth weight				X		
Complete physical examination of baby ^g^				X		
Limited physical examination of baby ^h^					X	
Maternal anthropometry: weight, height				X	X	
Child anthropometry: weight, length, head circumference				X	X	
Child vaccination and Vit A supplementation status					X	
Infant neurodevelopment using auditory brainstem responses (ABRs)					X	

^a^Complete examination: general appearance, throat, neck, thyroid, musculoskeletal, skin, lymph nodes, extremities, pulses, pulmonary, cardiac, abdominal, and neurological examination ^b^Limited examination: general appearance, brief pulmonary, cardiac, abdominal, and neurological examination ^c^e.g., Serum ferritin ^d^e.g., CRP ^e^Stool microbiome analysis ^f^Done only if mothers are still breast feeding ^g^Complete examination: weight, length, head circumference, APGAR score, Ballard score, new-born adiposity, congenital anomaly, and complications at birth ^h^Limited examination: general appearance, brief pulmonary, cardiac, abdominal, and neurological examination

### Trial design

REVAMP-TT is a two-arm open-label individual-randomized controlled trial in pregnant women with moderate or severe anemia (capillary Hb<10g/dL) during their third trimester (27-35 weeks gestation). Participants will be randomized to receive either parenteral iron – in the form of FCM– or standard-of-care oral iron. Women are recruited from health centers across Zomba district in Southern Malawi ( Figure 1). As REVAMP-TT is an effectiveness trial, the standard-of-care oral iron arm will be provided to participants following the established recommendations issued by the Malawi Ministry of Health. Screening of hemoglobin for eligibility and administration of the intervention ( *i.e.*, provision of the intravenous iron) takes place in government health centres. Mothers and their babies will be followed up to 12 months postpartum. The trial was registered with the Australia and New Zealand Clinical Trial Registry (ANZCTR 12621001239853) prior to the start of recruitment and followed the SPIRIT guidelines for randomized trials ^21^ throughout the design and reporting of this protocol.

### Study site and population

The trial is based at the Training and Research Unit of Excellence (TRUE) center at Zomba Central Hospital in Southern Malawi. The trial has eight government health centers across Zomba district available for screening, recruitment and provision of the intravenous drug as part of REVAMP-TT, namely: Likangala, Bimbi, Lambulira, Domasi, Naisi, Matawale, Sadzi and City clinic ( Figure 1). These facilities receive close to 1500 antenatal visits per month. All subsequent study visits will be done at TRUE center in Zomba.

Participants are eligible for the trial if they have a confirmed singleton pregnancy 27- to 35-weeks’ gestation either using last menstrual period or fundal height, have a capillary Hb concentration of less than 10 g/dL, measured by HemoCue 301+, and have negative malaria RDT. Additionally, the participants must be afebrile with no evidence of septicemia, reside and plan to give birth within the study catchment area of Zomba district and be able to provide written informed consent (or assent if <18 years old).

Women are excluded if they were previously enrolled in the REVAMP trial ^16, 18^ or are actively participating in another intervention trial. Women with known hypersensitivity to any of the study drugs, clinical symptoms of malaria or other infection, any condition requiring hospitalization, known history of sickle cell or sickle-hemoglobin C anemia, clinically unstable with a low hemoglobin level requiring a blood transfusion (usually Hb <5g/dL), or pre-eclampsia are excluded from participating in this trial. HIV positivity is not an exclusion.

### Randomization, treatment allocation and blinding

Using a randomization schedule of randomly permuted blocks stratified by site to achieve balance between the arms within each site, participants are randomly allocated 1:1 to one of the two treatment arms. An independent statistician computer-generated the randomization list, and sealed, opaque envelopes were used to randomly assign participants. Despite the trial being open-label, laboratory scientists measuring hemoglobin concentration, midwives collecting birth outcome data, and investigators and researchers at the Walter and Eliza Hall Institute, Melbourne, Australia (including data managers and statisticians) are all blinded to the treatment allocation throughout the trial duration until the database is locked and ready for unblinding.

### Study interventions and procedures

This trial takes place in primary health centers spread across Zomba district in Malawi, with several of the health centers considered to be remote – Likangala, Bimbi and Lambulira. ( Figure 1). As REVAMP-TT is delivered through government health centers using the available government health centers’ infrastructure, we used a co-design approach to develop interventions to support its implementation (REVAMP-IS study ^17^). Co-design is a two-staged approach to health systems and service improvements that typically involves an information-gathering stage followed by a second stage in which end users jointly develop the strategies with health service providers and the project leaders ^22^. Where the workload permits, government nurses directly administer the intervention and, in all clinics, government nurses received training and are encouraged to assist the trial team. Women in the intervention groups receive intravenous iron in the form of ferric carboxymaltose (Vifor Pharma) at a dosage of 20 mg/Kg body weight if <50 kg and a maximum of 1000 mg if body weight ≥50 kg given over 15 minutes in 250 mL of normal saline, once on recruitment day. The participant is observed for a minimum of 30 minutes before being discharged home. Women in the control group receive oral iron-200 mg ferrous sulphate (approx. 65 mg elemental iron) twice daily for the remainder of pregnancy. Importantly, the oral iron is given under real-life health service delivery conditions where the participant is given three months of oral iron at presentation to the antenatal clinic. This strategy is being employed as it is a component of the key hypothesis of this trial that there is a reduced effect of oral iron on maternal anemia due to poor adherence to the full course of treatment. In addition, all participants receive IPTp with SP, 1500 mg sulfadoxine and 75 mg pyrimethamine (three fixed tablets of SP strength at 500 mg/25 mg) as recommended in the national guidelines during study follow up visits ^23^. As part of the safety assessment, IPTp-SP post-randomization is directly observed by study staff.

All study activities at each study visit are summarized in Table 1.

### Data collection and management

Data are recorded in digital form with REDCap using electronic tablets and backed up nightly to a local backup server at TRUE, Blantyre, Malawi, with a de-identified fortnightly backup to the servers at the Walter and Eliza Hall Institute, Melbourne, Australia. REDCap is hosted on infrastructure belonging to the trial’s organizational team in Malawi and is subject to the same security and backup regimen as other systems ( *e.g.,* the network file servers).

## Study outcomes

### Primary outcome

The primary outcome is the proportion of women with anemia (defined as a venous blood hemoglobin < 11 g/dL) at 36 weeks gestation or delivery, whichever comes first. Gestational age in this setting is evaluated by last menstrual date and/or measurement of fundal height. These were chosen due to their common use in the government primary care centers where there is no availability of ultrasound, thus ensuring the generalizability of the results. 

### Secondary outcome(s)

Maternal secondary outcomes include hemoglobin, ferritin, anemia, iron deficiency, iron-deficiency anemia collected at 36 weeks gestation, delivery, and at one-month postpartum. Iron-deficiency is determined by measuring levels of serum ferritin and setting thresholds at ferritin<15ug/L or ferritin<30ug/L if C-reactive protein>5mg/L. Iron deficiency anemia indicates Hb<11g/dL and serum ferritin<15ug/L or ferritin<30ug/L if C-reactive protein>5mg/L ^24^. 

Neonatal outcomes include birth weight and low birth weight (birth weight <2,500g) within 24 hours of delivery and infant length-for-age z-score, weight-for-age z-score, and weight-for-length z-score as well as iron and hematological parameters collected at one-month postpartum.

### Safety outcomes

Safety outcomes for this trial include maternal and infant adverse events and severe adverse effects, hypophosphatemia (defined as Phosphate (PO4 <0.80 mmol/L), and inflammation status (threshold for inflammation set at C-reactive protein>5mg/L), measured at the same timepoints as specified for primary and secondary outcomes detailed above. 

## Sample size estimation and power calculation

The sample size for the trial was to recruit 590 pregnant women (295 per arm) to have 90% power to detect that FCM will result in a 14% reduction in absolute anemia prevalence compared with standard-of-care oral iron (49% to 63%) ^25^, allowing for a two-sided alpha of 5% and a 10% loss to follow up at the primary outcome. The sample size also has 72% to 97% power to detect an absolute difference between standard-of-care oral iron and FCM of 100 to 150g in the neonatal outcome of birth weight, assuming a standard deviation of 450g ^7^ and a two-sided alpha of 5%, after accounting for a miscarriage and stillbirth rate of 1%. Sample size calculations were performed using Stata/SE (StataCorp. 2019. College Station, TX: StataCorp LLC). The open-access software R can be used for sample size calculations where Stata/SE is unavailable.

The design incorporated an adaptive sample size re-estimation procedure based on the “promising zone” methodology of Mehta and Pocock ^26^ when the primary maternal outcome of at least 50% of the recruited participants were obtained. A sample size increase by up to 260 participants to the pre-specified maximum of 850 participants total (including loss to follow up) was allowed with the aim to achieve a conditional power of 90%, if the conditional power was in the “promising zone”. Using a one-tailed alpha of 0.025 and a power of 90%, the “promising zone” for conditional power was 0.388 to 0.9. The pre-planned sample size of 590 participants was confirmed as the final sample size after execution or the method by an independent unblinded statistician using the data from 77.8% (459/590) of the participants and recommendation from the Data Monitoring Committee. Sample size re-estimation was performed using a user-written program (Excel version 16.71).

## Statistical analysis and reporting

### Data Monitoring Committee

An independent Data Monitoring Committee (DMC) oversees the trial and is comprised of three international experts in clinical trials, obstetrics, epidemiology and statistics. The DMC recommends to the sponsor and investigators whether to continue, modify or terminate the trial on ethical grounds. The DMC evaluated the results of the sample size re-estimation (see sample size estimation and power calculation) and recommended that no sample size change was necessary.

### Statistical analysis principles / overview

Efficacy analyses will be performed according to the participants randomized treatment group and safety analyses will be performed according to the participants actual treatment group. Frequencies and percentages will be reported for categorical variables and mean and standard deviation or median and quartiles (25 ^th^ and 75 ^th^ percentile) for continuous variables. Treatment effects will be presented as point estimates with two-sided 95% confidence intervals (CIs). A two-sided P value <0.05 will be used to indicate statistical significance for the primary outcome and birth weight. The Holm procedure ^27^ will be used to control the family-wise error rate at 0.05 for maternal outcomes (hemoglobin concentration, and serum ferritin concentration at 36 weeks gestation or delivery (whichever comes first) and at one-month postpartum) and neonatal outcomes (hemoglobin concentration, serum ferritin concentration and weight at one-month of age) separately. Specifically, the Holm procedure is a stepwise multiple test method that controls the probability of one or more type 1 errors to be at most 5%, whereby null hypotheses are tested step-down using adjusted individual significance levels until no further rejections can be made ^26^. Analyses will be conducted using Stata/SE (StataCorp. 2019. College Station, TX: StataCorp LLC). The open-access software R can be used for sample size calculations where Stata/SE is unavailable.

### Main analysis of the primary outcome

A log-binomial regression model will be used to examine maternal anemia at the primary timepoint – 36 weeks’ gestation or delivery, whichever comes first – with study participants as a random intercept to account for multiple timepoints. A treatment and treatment-by-study-visit interaction and an adjustment for the stratification variable site (used during randomization) will be included in the model. The reference group for the model will be the standard-of-care oral iron treatment group. The prevalence ratio of IV iron versus standard-of-care oral iron will be estimated from this model as the treatment effect. In the event of non-convergence, we will use alternative models.

### Analysis of secondary outcome(s)

Identical to the analysis of the primary outcome repeated time-point binary outcomes (anemia, moderate/severe anemia, iron deficiency, iron deficiency anemia) will be analyzed. A likelihood-based longitudinal data analysis model ^28^ will be used to examine secondary repeated time-point continuous outcomes (hemoglobin concentration and ferritin concentration). Unstructured variance-covariance among the repeated measurements and a common baseline mean will be assumed by the model for the two treatment arms. The main effects of the model will be the stratification factor (site), the treatment and treatment by study visit interaction, and the study visit (timepoint) as a categorical variable. We will consider alternative structures (first-order autoregressive, Toeplitz, compound symmetry) in the event of non-convergence. The mean change between IV iron and standard-of-care oral iron will be used to estimate the treatment effect using this model. Before analysis, ferritin (μg/L) will be log _e_ transformed, and the treatment effect will be expressed as a geometric mean ratio. A linear regression model, adjusting for the stratification factor (site) will be used to analyze birth weight. The absolute difference in mean birth weight between IV iron and standard-of-care oral iron will be used to estimate the treatment effect based on this model.

Using the same analysis as used for birth weight, birth length within 24 hours of birth and hemoglobin concentration, ferritin concentration, length-for-age z-score, weight-for-age z-score, and weight-for-length z-score at one-month of age will be analyzed. If the variables are considered skewed, appropriate transformations may be applied to them prior to fitting the model. A log-binomial regression model, adjusting for the stratification factor (site), will be used to examine low birth weight and stillbirth. The risk ratio of IV iron versus standard-of-care oral iron will be estimated from this model as the treatment effect. In the event of non-convergence, we will use alternative models.

### Analysis of safety outcome(s)

Analyses of maternal and neonate adverse event outcomes, and neonate safety biomarkers (hypophosphatemia and malaria RDT positive) will be analyzed similarly to analyses of secondary single time-point binary outcomes detailed above. Maternal safety biomarkers (hypophosphatemia, inflammation and malaria RDT positive) will be analyzed using the same analysis as the primary maternal outcome (anemia). 

### Additional analyses

Using a model similar to the main analysis model of the primary outcome described above, the treatment effect at 36 weeks’ gestation will be examined for the primary outcome. Adjusted analyses for pre-specified covariates and analysis excluding participants with protocol violations (e.g., multiple births) will be included in additional analyses of primary, key secondary and other secondary outcomes. To determine the heterogeneity of the differences between IV iron and standard-of-care oral iron six pre-specified subgroup analyses (gravidity (primigravida vs. multigravida), baseline HIV status (positive vs negative), baseline severe anaemia status (yes vs no severe anaemia), baseline iron-deficient status (yes vs no ID), baseline iron-deficient anaemia status (yes vs no IDA), baseline inflammation status (yes vs no elevated CRP)) will be performed for primary and key secondary maternal and neonate outcomes. The missing-at-random assumption will be used to fit the model for the primary maternal outcome (anemia) and the missing-completely-at-random assumption will be used to fit the model for the primary neonate outcome (birth weight). Pattern-mixture models will be conducted to assess sensitivity of the results to these assumptions.

## Trial status

The trial started recruiting participants on the 24 ^th^ of November 2021 and completed enrolment on the 22 ^nd^ of February 2023. Follow-up visits for all participants were concluded in June 2023 for one-month postpartum and are expected to continue until May 2024, for one year postpartum.

## Ethics and dissemination

This protocol received the approval of the National Health Sciences Research Committee of Malawi Approval - NHSRC REF. Number: 20/11/2622, the Human Research Ethics committee at WEHI – HRE REF. Number: 20/25, and the Malawi Pharmacy and Medicines Regulatory Authority –PMRA/CTRC/III/08062021130.

Informed consent will be obtained from each participant before conducting any study related procedure. The information will be provided in the local language (Chichewa) of the participant and the participant will be given the opportunity to ask questions and allowed time to consider the information provided. If the participant is unable to write their signature, then a thumbprint may be used. If the participant is unable to read the information her/himself, full and comprehensive information will be communicated to the participant in the presence of a witness. Each original signed informed consent will be kept on file.

## Data handling and sharing

At the end of the study, the results will first be disseminated to national policymakers, government departments, academics from local research institutions and universities, National Health Sciences Research Committee, the College of Medicine Research and Ethics Committee and professional bodies in Malawi at the national stakeholders’ meeting or research dissemination conferences to be held in the country.

Research results will also be disseminated to the global research community, technical agencies, and international government bodies via peer-reviewed journals and at international scientific fora.

Study data will be made accessible under the terms of the Creative Commons Attribution 4.0 International license (CC-BY 4.0), comprising underlying de-identified individual participant data relevant to the reported trial results and a data dictionary.

## Discussion

The high prevalence of anemia during pregnancy in Africa, and specifically in Malawi, continues to be a major public health concern. This trial will provide the necessary evidence of the effectiveness of implementing intravenous iron for the treatment of anemia during pregnancy in the third trimester. The risk of adverse pregnancy outcomes due to anemia is particularly high in the third trimester, which is also the period when most pregnant women in LMICs present themselves for antenatal check-up. While IV iron has been used extensively in developed countries, this trial represents the first attempt to bring this intervention to primary health centers in LMICs. This trial will help determine whether Ferric Carboxymaltose represents an effective, safe, and feasible approach for the treatment of moderate to severe anemia in the third trimester of pregnancy, particularly in resource-limited countries such as Malawi.

If the trial demonstrates the effectiveness and safety of FCM in the third trimester, it could lead to a paradigm shift in the management of anemia during pregnancy in LMICs. By providing evidence-based guidance on the use of IV iron, this trial has the potential to improve maternal and fetal outcomes, reduce the risk of postpartum haemorrhage, and enhance infant health. Finally, the results of this trial will be useful in guiding policy decisions related to the use of IV iron in resource-limited settings, ultimately improving the overall health of pregnant women and their offspring in Malawi and other African countries.

## Data Availability

No data are associated with this article

## References

[1] WHO: The global prevalence of anaemia in 2011.Who,2011;1–48. Reference Source

[2] Haider BA Olofin I Wang M : Anaemia, prenatal iron use and risk of adverse pregnancy outcomes: Systematic review and meta-analysis. *BMJ.* 2013;346: f3443. 10.1136/bmj.f3443 23794316 PMC3689887

[3] Nair M Knight M Kurinczuk J : Risk factors and newborn outcomes associated with maternal deaths in the UK from 2009 to 2013: a national case-control study. *BJOG.* 2016;123(10):1654–62. 10.1111/1471-0528.13978 26969482 PMC5021205

[4] Drassinower D Lavery JA Friedman AM : The effect of maternal haematocrit on offspring iq at 4 and 7 years of age: A secondary analysis. *BJOG.* 2016;123(13):2087–93. 10.1111/1471-0528.14263 27533357

[5] WHO: Global nutrition targets 2025: Anaemia Policy Brief.2012;1–7. Reference Source

[6] Peña-Rosas JP De-Regil LM Garcia-Casal MN : Daily oral iron supplementation during pregnancy. *Cochrane Database Syst Rev.* 2015;2015(7): CD004736. 10.1002/14651858.CD004736.pub5 26198451 PMC8918165

[7] Mwangi MN Roth JM Smit MR : Effect of daily antenatal iron supplementation on plasmodium infection in kenyan women: A randomized clinical trial. *JAMA.* 2015;314(10):1009–20. 10.1001/jama.2015.9496 26348751

[8] WHO: Iron and Folate Supplementation: Intergrated Management of Pregnancy and Childbirth (IMPAC).Geneva,2006.

[9] WHO: Essential Nutrition Actions: improving maternal, newborn, infant and young child health and nutrition.Geneva,2013. Reference Source 25473713

[10] Low MSY Speedy J Styles CE : Daily iron supplementation for improving anaemia, iron status and health in menstruating women. *Cochrane Database Syst Rev.* 2016;4(4): CD009747. 10.1002/14651858.CD009747.pub2 27087396 PMC10182438

[11] Bah A Pasricha SR Jallow MW : Serum Hepcidin Concentrations Decline during Pregnancy and May Identify Iron Deficiency: Analysis of a Longitudinal Pregnancy Cohort in The Gambia. *J Nutr.* 2017;147(6):1131–7. 10.3945/jn.116.245373 28424258 PMC5443464

[12] Friedrisch JR Cançado RD : Intravenous ferric carboxymaltose for the treatment of iron deficiency anemia. *Rev Bras Hematol Hemoter.* 2015;37(6):400–5. 10.1016/j.bjhh.2015.08.012 26670403 PMC4678908

[13] Qassim A Grivell RM Henry A : Intravenous or oral iron for treating iron deficiency anaemia during pregnancy: systematic review and meta‐analysis. *Med J Aust.* 2019;211(8):367–73. 10.5694/mja2.50308 31441077

[14] Pollock RF Muduma G : A systematic literature review and indirect comparison of iron isomaltoside and ferric carboxymaltose in iron deficiency anemia after failure or intolerance of oral iron treatment. *Expert Rev Hematol.* 2019;12(2):129–36. 10.1080/17474086.2019.1575202 30689458

[15] National Health Service: Ferinject in Pregnancy and the Postpartum Period.2018. Reference Source

[16] Pasricha SR Mwangi MN Moya E : Ferric carboxymaltose versus standard-of-care oral iron to treat second-trimester anaemia in Malawian pregnant women: a randomised controlled trial. *Lancet.* 2023;401(10388):1595–1609. 10.1016/S0140-6736(23)00278-7 37088092 PMC10193370

[17] Prang KH Mamani-Mategula E Verbunt E : An implementation research programme to support an intravenous iron intervention for pregnant women with moderate and severe anaemia in Malawi: study protocol. *Implement Sci Commun.* 2022;3(1): 68. 10.1186/s43058-022-00299-x 35729604 PMC9210048

[18] Mwangi MN Mzembe G Moya E : Protocol for a multicentre, parallel-group, open-label randomised controlled trial comparing ferric carboxymaltose with the standard-of-care in anaemic Malawian pregnant women: the REVAMP trial. *BMJ Open.* 2021;11(11): e053288. 10.1136/bmjopen-2021-053288 34815287 PMC8611444

[19] Harding R Ataide R Mwangi MN : A Randomized controlled trial of the Effect of intraVenous iron on Anaemia in Malawian Pregnant women (REVAMP): Statistical analysis plan [version 2; peer review: 2 approved]. *Gates Open Res.* 2022;5:174. 10.12688/gatesopenres.13457.2 35492865 PMC9019159

[20] Bothwell TH : Iron requirements in pregnancy and strategies to meet them. *Am J Clin Nutr.* 2000;72(1 Suppl):257S–264S. 10.1093/ajcn/72.1.257S 10871591

[21] Chan AW Tetzlaff JM Gøtzsche PC : SPIRIT 2013 explanation and elaboration: guidance for protocols of clinical trials. *BMJ.* 2013;346: e7586. 10.1136/bmj.e7586 23303884 PMC3541470

[22] Richard L Piper D Weavell W : Advancing engagement methods for trials: The CORE study relational model of engagement for a stepped wedge cluster randomised controlled trial of experience-based co-design for people living with severe mental illnesses. *Trials.* 2017;18(1): 169. 10.1186/s13063-017-1878-7 28388937 PMC5385022

[23] Ministry of Health Malawi: Malawi National Reproductive Health Service Delivery Guidelines 2014-2019. Reference Source

[24] Pasricha SR Hasan MI Braat S : Benefits and Risks of Iron Interventions in Infants in Rural Bangladesh. *N Engl J Med.* 2021;385(11):982–995. 10.1056/NEJMoa2034187 34496174

[25] Breymann C Milman N Mezzacasa A : Ferric carboxymaltose vs. oral iron in the treatment of pregnant women with iron deficiency anemia: An international, open-label, randomized controlled trial (FER-ASAP). *J Perinat Med.* 2017;45(4):443–453. 10.1515/jpm-2016-0050 27278921

[26] Mehta CR Pocock SJ : Adaptive increase in sample size when interim results are promising: A practical guide with examples. *Stat Med.* 2011;30(28):3267–84. 10.1002/sim.4102 22105690

[27] Holm S : A Simple Sequentially Rejective Multiple Test Procedure. 1979. Reference Source

[28] Liang KY Zeger SL : Longitudinal Data Analysis of Continuous and Discrete Responses for Pre-Post Designs. 1960. Reference Source

