# Supplementary material for: Protocol and statistical analysis plan for a randomized controlled trial of the effect of intravenous iron on anemia in Malawian pregnant women in their third trimester (REVAMP – TT)
Source: Gates Open Res. 2023 Dec 18;7:117. Originally published 2023 Sep 5. [Version 2] doi: 10.12688/gatesopenres.14710.2 (PMC10858019; doi:10.12688/gatesopenres.14710.2)
Supplement: Peer review history

# Reviewer report: Reviewer response for version 2

**DOI:** 10.21956/gatesopenres.16541.r35686

**Published:** 2024-02-09

**Authors:** Sue Pavord

**Affiliations:** 1 Department of Haematology, Oxford University Hospitals NHS Foundation Trust, Oxford, UK

Thank you for the responses concerning the oral iron arm. I appreciate that the aim is to compare intravenous iron with how oral iron is currently used in Malawi.

 I approve the study on this basis. However I would like to register that, as this is not the optimal way to use oral iron (too high a dose and inadequate counseling) this is a missed opportunity to help determine whether resources are better used for the provision of intravenous iron (for malaria-negative women with unclassified anaemia), or used to ensure oral iron is administered correctly, to optimise absorption and compliance. I believe this to be an important question for all countries where resources need to be used judiciously.

Is the study design appropriate for the research question?

Yes

Is the rationale for, and objectives of, the study clearly described?

Yes

Are sufficient details of the methods provided to allow replication by others?

Yes

Are the datasets clearly presented in a useable and accessible format?

Yes

Reviewer Expertise:

Haematology, pregnancy, iron management

I confirm that I have read this submission and believe that I have an appropriate level of expertise to confirm that it is of an acceptable scientific standard.

# Reviewer report: Reviewer response for version 1

**DOI:** 10.21956/gatesopenres.16036.r35087

**Published:** 2023-10-06

**Authors:** Sue Pavord

**Affiliations:** 1 Department of Haematology, Oxford University Hospitals NHS Foundation Trust, Oxford, UK

Thank you for asking me to review this protocol.

 The study is well thought out and planned. The inclusion and exclusion criteria are realistic, pragmatic and ideal.

 My concern is the oral iron arm. 

 Firstly for optimal absorption, ferrous sulphate should not be given more than once a day. More successive medication impairs absorption and causes side effects due to increased unabsorbed iron in the gut lumen.

 Furthermore it should be emphasised that the iron tablet needs to be taken early in the morning (when hepcidin levels are low), with a glass of water or freshly squeezed orange juice, on an empty stomach, one hour before tea/coffee/breakfast and any other medications or multivitamins (all of which would interfere with iron absorption).

 I also note the authors' decision not to monitor adherence to oral iron. This was how it was done in the earlier REVAMP trial and has merit in reflecting the real-world scenario. However this time perhaps the opportunity could be taken to see how oral iron can be effective if it is taken properly. Monitoring would be easy to achieve in the trial as it would coincide with monitoring of  SP administration. 

 If oral iron is then found to be effective in this trial, it can be debated whether resources should be allocated to counselling re correct administration and improving adherence to oral iron, or allocated to facilities and systems for intravenous iron administration. i.e. oral iron needs to be given a fair chance in this trial.

Is the study design appropriate for the research question?

Yes

Is the rationale for, and objectives of, the study clearly described?

Yes

Are sufficient details of the methods provided to allow replication by others?

Yes

Are the datasets clearly presented in a useable and accessible format?

Yes

Reviewer Expertise:

Haematology, pregnancy, iron management

I confirm that I have read this submission and believe that I have an appropriate level of expertise to confirm that it is of an acceptable scientific standard, however I have significant reservations, as outlined above.

# Response

**Published:** 2023-12-13

**Authors:** Ricardo Ataide

**Affiliations:** Population Health and Immunity, Walter and Eliza Hall Institute of Medical Research, Melbourne, Victoria, Australia

We would like to thank all the reviewers for the time taken to carefully read through the manuscript and help us improve it. We have replied to each reviewer independently and highlighted the changes made to the manuscript (where needed) in black bold italic text. 

**Reviewer 2 – Sue Pavord, Oxford University Hospitals NHS Foundation Trust, Oxford, UK**

 Thank you for the positive feedback on our trial protocol and statistical analysis plan. We understand that the main comments are related to the Oral iron arm of the trial. We have addressed those comments below and combined some of them together as the answers are very similar.

  

1. Firstly for optimal absorption, ferrous sulphate should not be given more than once a day. More successive medication impairs absorption and causes side effects due to increased unabsorbed iron in the gut lumen.
2. Furthermore it should be emphasised that the iron tablet needs to be taken early in the morning (when hepcidin levels are low), with a glass of water or freshly squeezed orange juice, on an empty stomach, one hour before tea/coffee/breakfast and any other medications or multivitamins (all of which would interfere with iron absorption).

In REVAMP-TT, as in our previous REVAMP trial of IV iron during the second trimester of pregnancy, the Oral iron arm is intended to reflect standard-of-care in Malawi. The Obstetrics and Gynaecology Protocol and Guideline (2017) and the Malawi Standard Treatment Guideline (2023, p.251) recommends Ferrous Sulphate/Folic acid 200mg 12 hourly as treatment for asymptomatic moderate/severe (not needing blood transfusion) anaemia in pregnancy. Ferrous Sulphate 200mg once is given to all antenatal women in Malawi as per WHO and Malawi national guidelines.

 We agree that iron tablets need to be given early in the morning with a glass of water, on an empty stomach and this information was given to the participants. We did not include information on one hour before tea/coffee as providing this information could be deviating from the information that is given routinely in this setting.

  

1. I also note the authors' decision not to monitor adherence to oral iron.
  1. This was how it was done in the earlier REVAMP trial and has merit in reflecting the real-world scenario. However this time perhaps the opportunity could be taken to see how oral iron can be effective if it is taken properly.
  2. Monitoring would be easy to achieve in the trial as it would coincide with monitoring of SP administration.

We appreciate the reviewer’s interest in quantifying the outcome for pregnant women were they to receive and take Oral iron as recommended to achieve optimal iron levels. The main reasons for not monitoring adherence in the Oral iron group are:

  

1. Previous trials in similar settings done in pregnancy have evaluated how, in a clinical research setting, a constantly monitored population responds positively to oral iron supplementation ^2^. The goal of the REVAMP-TT trial is effectiveness, rather than efficacy, of IV Iron compared to Oral iron. For that purpose, the two interventions need to be given in as ‘real-world’ scenario as possible. We have included the following text to emphasise this, under Trial Design (page 6):

*“REVAMP-TT is a two-arm open-label individual-randomized controlled trial in pregnant women with moderate or severe anemia (capillary Hb <10g/dL) during their third trimester (27–35 weeks gestation). Participants will be randomized to receive either parenteral iron – in the form of FCM– or standard-of-care oral iron. **As REVAMP-TT is an effectiveness trial, the standard-of-care oral iron arm will be provided to participants following the established recommendations issued by the Malawi Ministry of Health.**”*

  

1. Due to the disparate nature of IPTp-SP dosage and administration (i.e., three tablets under direct observation) versus the oral iron administration and dosage (i.e., two tablets daily for duration of pregnancy), it would be difficult to monitor them concurrently.
2. If oral iron is then found to be effective in this trial, it can be debated whether resources should be allocated to counselling re correct administration and improving adherence to oral iron, or allocated to facilities and systems for intravenous iron administration. i.e. oral iron needs to be given a fair chance in this trial.

We agree with the reviewer that understanding the non-biological factors that influence the outcome of an intervention in a specific geographical and cultural setting is key to the success of that intervention. As the trial is designed to assess IV Iron and its implementation in Malawi in a ‘real-world’ scenario, we are conducting – concurrently with REVAMP-TT – a research implementation programme that evaluates the contextual factors that facilitate or impede that intervention. This is noted on page 7:

*﻿“As REVAMP-TT is delivered through government health centers using the available government ﻿health centers’ infrastructure, we used a co-design approach to develop interventions to support its implementation (REVAMP-IS study ^17^). Co-design is a two-staged approach to health systems and service improvements that typically involves an information-gathering stage followed by a second stage in which end users jointly develop the strategies with health service providers and the project leaders ^23^.”*

 References:

 1.        Pasricha, S.-R. *et al.* Ferric carboxymaltose versus standard-of-care oral iron to treat second-trimester anaemia in Malawian pregnant women: a randomised controlled trial. *Lancet* **401**, 1595–1609 (2023).

 2.        Mwangi, M. N. *et al.* Effect of daily antenatal iron supplementation on plasmodium infection in kenyan women: A randomized clinical trial. *JAMA - Journal of the American Medical Association* **314**, 1009–1020 (2015).

# Reviewer report: Reviewer response for version 1

**DOI:** 10.21956/gatesopenres.16036.r35088

**Published:** 2023-10-05

**Authors:** Adam Lewkowitz

**Affiliations:** 1 Department of Obstetrics and Gynecology, Women and Infants Hospital of Rhode Island, Brown University, Providence, Rhode Island, USA

The authors are to commended for completing another high-quality randomized trial in Malawi, the results of which may transform care delivery in this country and others.  Given recruitment ended  February, 2023 and final follow-up visits finished in June, 2023, the decision to publish the study protocol now reduces the ability of reviewers or editors to provide feedback which may be incorporated to strengthen the trial. As is, there are two additional limitations to this study that may be mitigated should the authors elect to conduct additional stratified analyses:  1) the heterogeneous primary aim; and 2) the lack of confirmation of iron deficiency anemia (IDA) before randomization. For the heterogeneous outcome, the potential influence that delivery prior to 36 weeks may have on the primary outcome can be mitigated by conducting a stratified analysis limiting to only those who delivered at 36 weeks. For the lack of IDA at delivery, the authors note that "serum for iron markers tests" will be drawn at enrollment. Thus, it may be possible to limit analysis only to those who had confirmed IDA by ferritin or other iron studies (with whatever ferritin threshold they elect to use).

Is the study design appropriate for the research question?

Yes

Is the rationale for, and objectives of, the study clearly described?

Yes

Are sufficient details of the methods provided to allow replication by others?

Yes

Are the datasets clearly presented in a useable and accessible format?

Yes

Reviewer Expertise:

Iron deficiency anemia, pregnancy, health services research

I confirm that I have read this submission and believe that I have an appropriate level of expertise to confirm that it is of an acceptable scientific standard.

# Response

**Published:** 2023-12-13

**Authors:** Ricardo Ataide

**Affiliations:** Population Health and Immunity, Walter and Eliza Hall Institute of Medical Research, Melbourne, Victoria, Australia

We would like to thank all the reviewers for the time taken to carefully read through the manuscript and help us improve it. We have replied to each reviewer independently and highlighted the changes made to the manuscript (where needed) in black bold italic text. 

**Reviewer 1 – Adam Lewkowitz, Brown University, Providence, USA**

 Thank you for your positive feedback and endorsement of our manuscript. We have addressed your comments below:

  

1. The heterogeneous primary aim; (…) the potential influence that delivery prior to 36 weeks may have on the primary outcome can be mitigated by conducting a stratified analysis limiting to only those who delivered at 36 weeks.

Thank you for this suggestion. To explore the potential influence of delivery prior to 36 weeks’ gestation on the primary outcome and thus provide further insight into the treatment effect at 36 weeks’ gestation, we propose conducting an additional analysis by analysing the outcome data at the 36 weeks’ gestation visit for all randomised participants. In this analysis, the statistical model will use outcome data at the 36 weeks’ gestation visit for participants who did not deliver prior to or at this visit. For participants who delivered prior to or at 36 weeks’ gestation, no outcome data at 36 weeks’ gestation during pregnancy could be obtained and the statistical model will consider the outcome data as missing at the 36 week’s gestation visit. The following bold italic text has been added/amended in the additional analyses section on page 16:

*“ **Using a model similar to the main analysis model of the primary outcome described above, the treatment effect at 36 weeks’ gestation will be examined for the primary outcome. **Adjusted analyses for pre-specified covariates and analysis excluding participants with protocol violations (e.g., multiple births) will be included in additional analyses of primary, key secondary and other secondary outcomes. To determine the heterogeneity of the differences between IV iron and standard-of-care oral iron six pre-specified subgroup analyses will be performed for primary and key secondary maternal and neonate outcomes. The missing-at-random assumption will be used to fit the model for the primary maternal outcome (anemia) and the missing-completely-at-random assumption will be used to fit the model for the primary neonate outcome (birth weight). Pattern-mixture models will be conducted to assess sensitivity of the results to these assumptions.”*

1. The lack of confirmation of iron deficiency anemia (IDA) before randomization. (…) the authors note that "serum for iron markers tests" will be drawn at enrolment. Thus, it may be possible to limit analysis only to those who had confirmed IDA by ferritin or other iron studies (with whatever ferritin threshold they elect to use).

We agree that IDA before randomisation may be predictive of the effect of IV iron. Therefore, a pre-planned subgroup analysis for IDA will explore the heterogeneity of treatment effect between those with and without IDA at baseline. We have amended the text to more explicitly state our pre-specified subgroup analyses. It now reads as follows, under ‘Additional Analyses’ in page 17:

*“To determine the heterogeneity of the differences between IV iron and standard-of-care oral iron six pre-specified subgroup analyses **(gravidity (primigravida vs. multigravida), baseline HIV status (positive vs negative), baseline severe anaemia status (yes vs no severe anaemia), baseline iron-deficient status (yes vs no ID), baseline iron-deficient anaemia status (yes vs no IDA), baseline inflammation status (yes vs no elevated CRP))** will be performed for primary and key secondary maternal and neonate outcomes.”*
